# Supplementary material for: ATP6V0A1 encoding the a1-subunit of the V0 domain of vacuolar H+-ATPases is essential for brain development in humans and mice
Source: Nat Commun. 2021 Apr 8;12:2107. doi: 10.1038/s41467-021-22389-5 (PMC8032687; doi:10.1038/s41467-021-22389-5)
Supplement: Supplementary file 1 — Supplementary Information [file 41467_2021_22389_MOESM1_ESM.pdf]

Supplementary information

ATP6V0A1 encoding the a1-subunit of the V0 domain of vacuolar H^+-ATPases is essential for brain development in humans and mice

Kazushi Aoto, Mitsuhiro Kato, Tenpei Akita, Mitsuko Nakashima, Hiroki Mutoh, Noriyuki Akasaka, Jun Tohyama, Yoshiko Nomura, Kyoko Hoshino, Yasuhiko Ago, Ryuta Tanaka, Orna Epstein, Revital Ben-Haim, Eli Heyman, Takehiro Miyazaki, Hazrat Belal, Shuji Takabayashi, Chihiro Ohba, Atsushi Takata, Takeshi Mizuguchi, Satoko Miyatake, Noriko Miyake, Atsuo Fukuda, Naomichi Matsumoto and Hirotomo Saitsu

1. Supplementary text
2. Supplementary Figures 12
3. Supplementary Tables 2

Case reports

Individual 1

Individual 1 was spontaneously born at 40 weeks of gestation with a coiling of the umbilical cord but no asphyxia as the first child of unrelated healthy parents. Her mother noticed that the individual showed poor sucking and little crying after birth, but medical stuffs disagreed it. She showed social smile at 1-2 months of age and head control at 5 months of age. At the age of 7 months, she had the first seizure showing munching and staring with touching or handling her own arms for 10 to 20 seconds. The frequency of her seizure rapidly increased up to 10 times a day. Valproic acid was prescribed but it was ineffective even after the dose was maximized. Her seizures were difficult to control and her psychomotor development was gradually delayed. She rolled-over at 12 months, sat alone at 11 months, crawled at 11 to 12 months, and stood alone at 15 months. Her seizures temporarily disappeared with a combination of phenobarbital, vitamin B6, phenytoin, and zonisamide at 17 months. She walked alone at 22 months. At 2 years and 10 months of age, EEG showed spikes or polyspikes. After then, epileptic discharges on EEG disappeared. She spoke meaningful words at 3 years and 6 months. At 3 years and 9 months of age, a status of clonic convulsion with right facial twitching and ocular supraduction continued for 50 minutes during sleep state under a medication of theophylline. Then, recurrent episodes of clonic convulsion occurred every two months. Another episode of convulsive status for more than 4 hours happened two days after her discharge from the hospital due to influenza infection at 4 years and 8 months. She showed focal impaired awareness seizure with or without vomiting for 10 to 20 seconds at 5 years. She was medicated with vitamin B6, phenytoin, zonisamide, and primidone, but her seizure occurred daily. At 6 years of age, her height was 106 cm (-0.8 SD), body weight 17.3 kg (-1.5 SD), head circumference 46.6 cm (-2.4 SD).

Individual 2

Individual 2 was delivered by vacuum extraction due to weak labor pain and obstructed labor at 36 weeks of gestation as a dizygotic twin after *in vitro* fertilization. He spent a day in an incubator and received phototherapy for jaundice. His family history was unremarkable except for maternal grandfather's brother showing intellectual disability. He did not show eye contact at 5 months of age and visual impairment was pointed out. His eye movement was saccadic. His motor skill was a level of 3-4 months old and his intellectual level was 1-2 months old at that time. He had epileptic spasms in cluster with hypsarrhythmia on EEG at 6 months. An administration of vitamin B6 was ineffective for epileptic spasms and he showed tonic seizures as well. Laboratory examinations including chromosomal karyotype and cerebrospinal fluid test were unremarkable. His seizures were refractory to valproic acid, but adrenocorticotropic hormone injection therapy abolished epileptic spasms. He still had tonic-clonic seizures, which disappeared after zonisamide administration. Zonisamide was replaced with clobazam because of anorexia at 10 months of age. He could walk alone at 1 year but showed a tendency of hyperactivity with no meaningful words at 4 years. He kept seizure-free state and anti-epileptic medications finished at 7 years for a time. At the age of 10 years, tonic-clonic seizures relapsed after tumbling and getting a blow on the head. Valproic acid, levetiracetam and topiramate were unsuccessful and he still had focal impaired awareness seizure once or twice a month. He was unable to record EEG since 8 years because of no cooperation. At the age of 14 years, he showed profound intellectual disability, no meaningful words, autistic behavior, hyperactivity, agitation, and self-injury with patting his head. His DQ score was 10 by Japanese Enjoji developmental scale test.

Individual 3

Individual 3 was born by cesarean section due to the last time for cesarean section at 36 weeks of gestation after polyhydramnios since 26 weeks of gestation. He was the fourth child of unrelated healthy parents. No asphyxia was observed with Apgar scores 8 and 9 at one and five minutes,

respectively, but he showed salivation and feeding difficulty after birth. He was transferred to the hospital due to obstructive apneic spells at 1 day after birth. He started tube feeding because he could not suck and swallow. He also needed endotracheal intubation due to aspiration pneumonia at 9 days of age. His EEG showed suppression burst composed by periodic epileptic discharges at occipitotemporal area with left-side predominance within 4 seconds and almost completely flat phase lasting 22 seconds at longest. Brain MRI at 10 days of age showed cortical dysplasia and cerebellar hypoplasia. Anticonvulsants, such as phenobarbital, clobazam and valproic acid, were administered but no improvement was seen on EEG. Subtle seizures, such as pedaling and rowing of the extremities, appeared at 27 days, then epileptic spasms on bilateral upper extremities started at 29 days. His seizures were intractable and he showed severe developmental delay, recurrent aspiration pneumonia, and gastroesophageal reflux, which needed tracheostomy at 2 months, gastrostomy and fundoplication at 6 months, and laryngotracheal separation at 8 months of age. He also showed bone fracture owing to osteoporosis and malnutrition because of frequent diarrhea. At 6 years 11 months, he showed spastic quadriplegia, systemic joint contractures, bed-ridden state, no eye contact, and no social smile. His seizures were daily erratic myoclonus during awake state, which was refractory to zonisamide, levetiracetam, potassium bromide, and clonazepam. EEG showed low-amplitude background activity with no epileptic discharges.

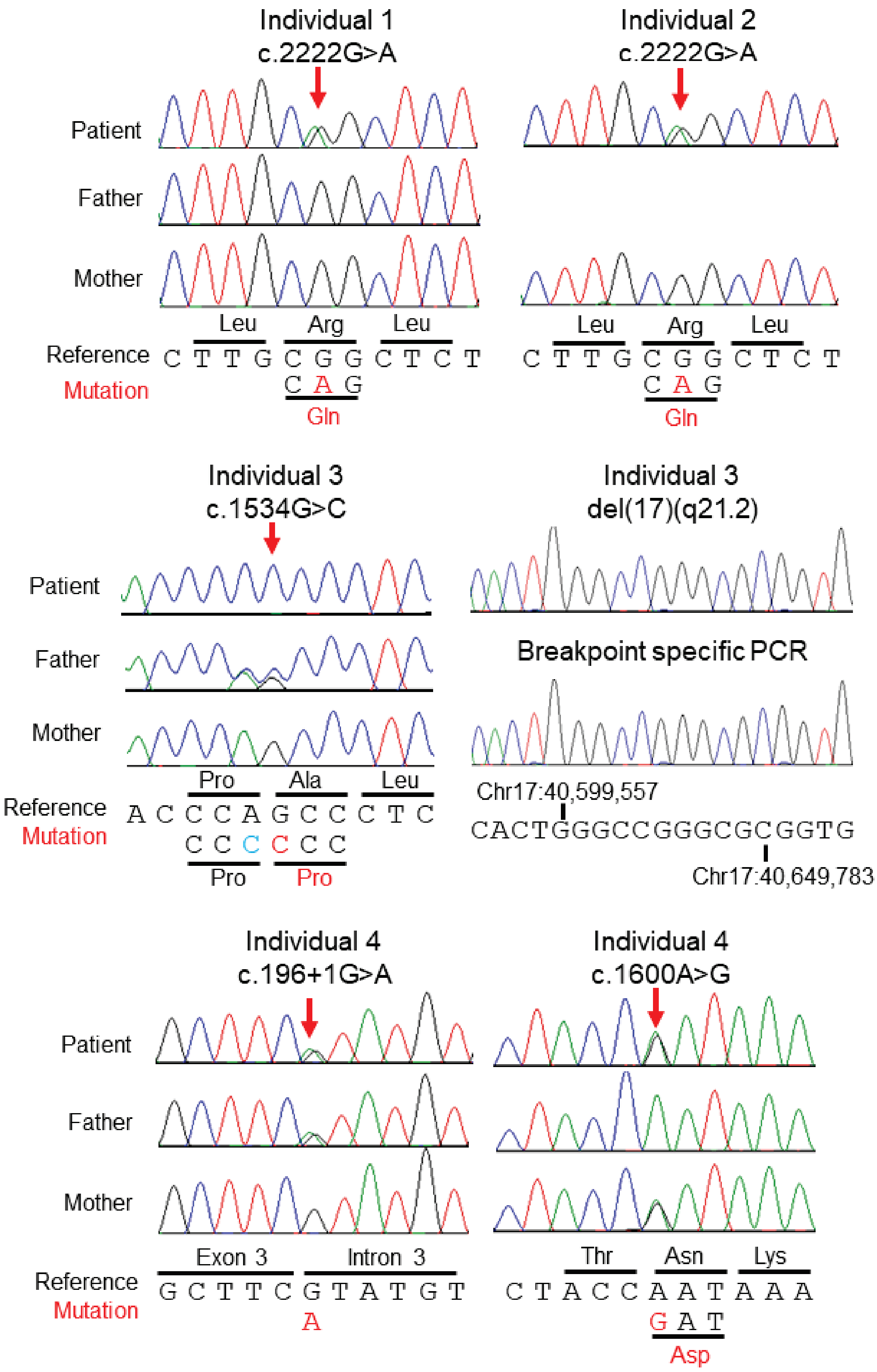

Supplementary Fig. 1 Sanger sequencing with available family members showing segregation of *ATP6V0A1* variants.

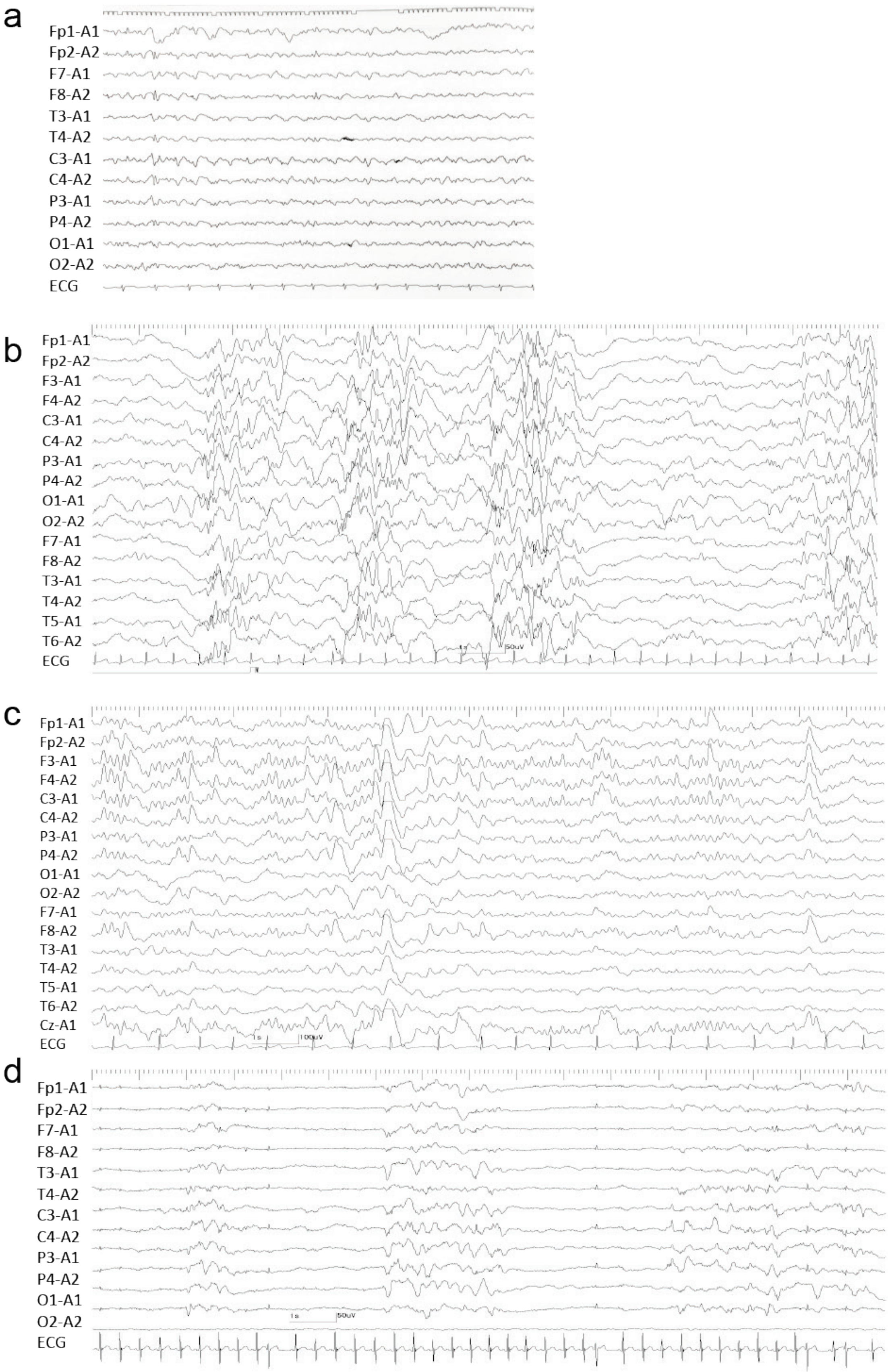

Supplementary Fig. 2 Electroencephalogram (EEG) of the individuals with *ATP6V0A1* variant. Individual 1 at 18 years of age shows focal or hemispherical epileptic discharges mainly at F8 (**a**). Individual 2 shows periodic pattern of high-amplitude diffuse or multifocal epileptic

discharges mixed with irregular slow waves compatible to hypsarrhythmia at 6 months of age (**b**), then it normalized at 7 years of age (**c**). EEG of individual 3 at 8 months shows an intermittent pattern composed by the periods of almost flat activity and the periods of 50-100 microvolts slow-waves with multifocal epileptic discharges (**d**). This pattern is similar to suppression-burst, but the amplitude of the burst phase is lower than that usually seen in the individuals with Ohtahara syndrome or early myoclonic encephalopathy.

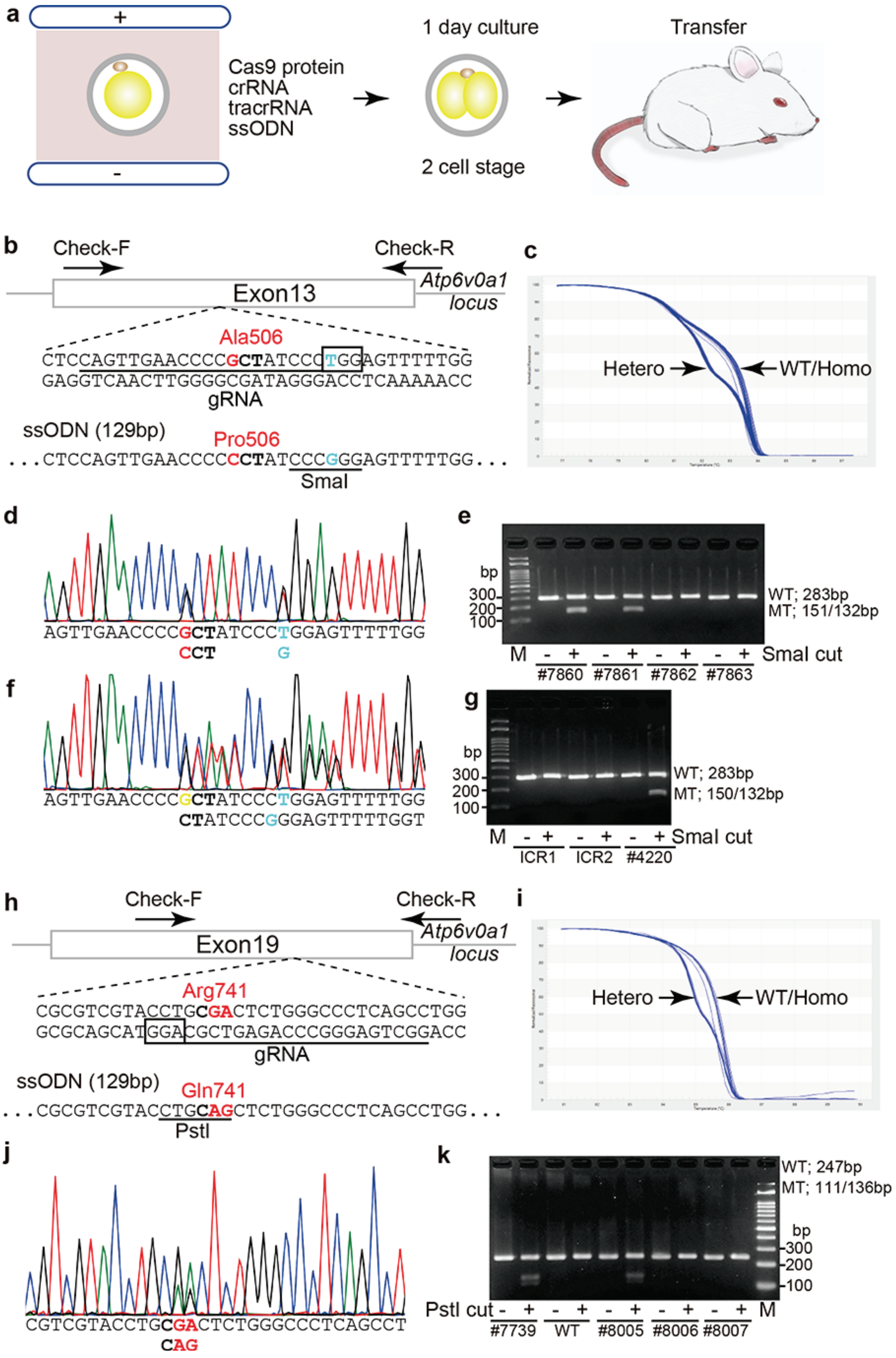

Supplementary Fig. 3 Generation of *Atp6v0a1*^{A512P} and *Atp6v0a1*^{R741Q} mutant mice. (**a**) The diagram of the 1-mm gap electroporation method. Left, ribonucleoprotein (Cas9 protein, crRNA,

tracrRNA) and ssODN solution are filled in the 1-mm gap electrode. Middle, electroporated one-cell stage eggs are developed into two-cell stage eggs. Right, developed eggs are transferred into the oviduct ampulla of host mother mice. (**b**, **h**) Schematic representation of targeting sites of the mouse *Atp6v0a1*^{A512P} locus with a SmaI recognition site (**b**) and the *Atp6v0a1*^{R741Q} locus with a PstI recognition site (**h**). Black line boxes indicate the protospacer adjacent motif (PAM) sequences. Black underlines show 20-nucleotide guide RNA targeting sequences. Red colored bases show the changed nucleotides. The T to G change highlighted in blue color was made for creating the recognition site of SmaI restriction enzyme (**b**). (**c**, **i**) High resolution melting analysis for grouping heterozygous and wild-type (WT) or *Atp6v0a1*$^{A512P/A512P}$ (**c**) or *Atp6v0a1*$^{R741Q/R741Q}$ (**i**) homozygous pups. (**d**, **f**, **j**) Direct sequencing of PCR products derived from the *Atp6v0a1*$^{A512P/+}$ (**d**), *Atp6v0a1*$^{KO/+}$ (**f**) and *Atp6v0a1*$^{R741Q/+}$ (**j**) mutant mice. (**e**, **g**, **k**) Genotyping of *Atp6v0a1*$^{A512P/+}$ (**e**), *Atp6v0a1*$^{KO/+}$ (**g**) and *Atp6v0a1*$^{R741Q/+}$ (**k**) mutant mice by restriction enzyme digestion. WT, wild-type; MT, mutant. Data in **e**, **g** and **k** are representative of two independent experiments. Source data are provided as a Source Data file.

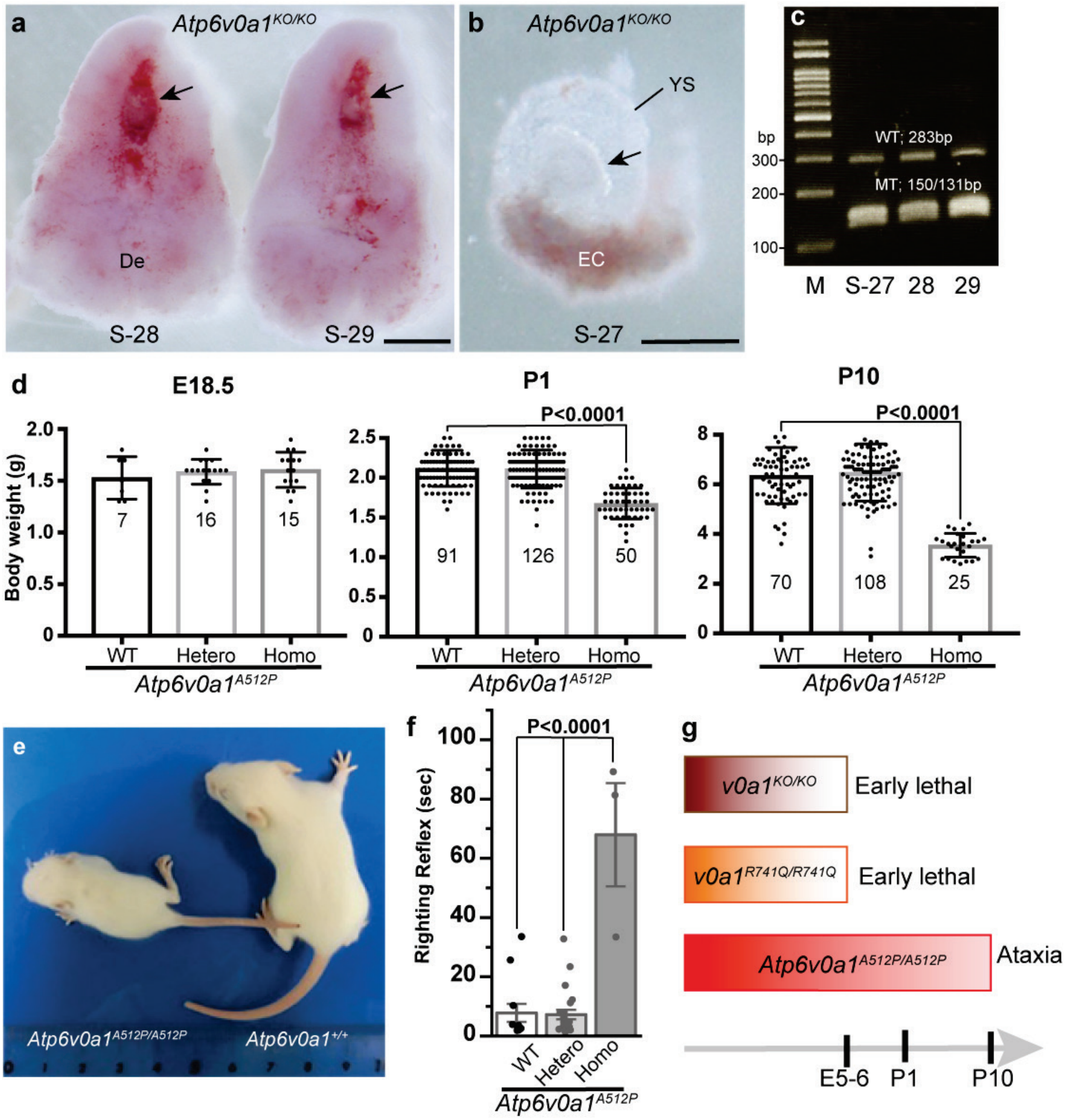

Supplementary Fig. 4 Survival period of three *Atp6v0a1* mutant mice lines. (**a**) The morphology of *Atp6v0a1*$^{KO/KO}$ embryos at embryonic day 7.5 (E7.5). Arrows show dead embryos inside decidua (De). Scale bars, 0.5 mm. (**b**) Development of *Atp6v0a1*$^{KO/KO}$ embryos stopped at around E5-6 of two layer stage (arrow). Scale bars, 100 µm. (**c**) Genotyping of dead embryos, indicating that all three dead embryos were homozygous (*Atp6v0a1*$^{KO/KO}$). Abbreviation: YS, yolk sac; EC, ectoplacental cone. (**d**) Body weight of *Atp6v0a1*$^{+/+}$ (WT), *Atp6v0a1*$^{A512P/+}$ (Hetero) and *Atp6v0a1*$^{A512P/A512P}$ (Homo) mutants at E18.5, P1 and P10. *Atp6v0a1*$^{A512P/A512P}$ mutant mice at P1 and P10 showed significantly reduced body weight, but not at E18.5. Sample numbers analyzed are

shown in bars. (**e**) *Atp6v0a1*$^{A512P/A512P}$ pups are smaller than wild-type pups at the same age. (**f**) Righting reflex in *Atp6v0a1*^{A512P} pups at P3. The time taken to right themselves from a supine position was significantly increased in *Atp6v0a1*$^{A512P/A512P}$ pups (WT n = 7, Hetero n=23, Homo n=3). (**g**) Survival periods and phenotypes of *Atp6v0a1*$^{KO/KO}$, *Atp6v0a1*$^{A512P/A512P}$ and *Atp6v0a1*$^{R741Q/R741Q}$ mice. *Atp6v0a1*$^{KO/KO}$ and *Atp6v0a1*$^{R741Q/R741Q}$ embryos died around E5-6. Ordinary one-way ANOVA with Dunnett's multiple comparison test was used for comparing WT with *Atp6v0a1*$^{A512P/A512P}$pups (**d**, **f**). Data represented as mean values ± standard deviation (**d**, **f**). Embryo morphology and genotyping data in **a**, **b** and **c** are representative of two independent experiments. Source data are provided as a Source Data file.

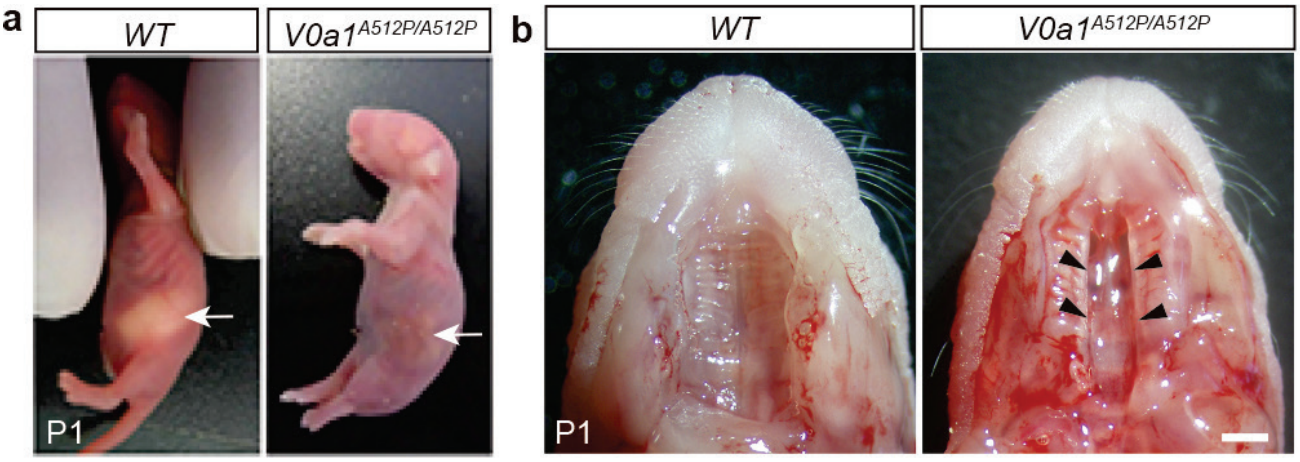

Supplementary Fig. 5 ***Atp6v0a1*$^{A512P/A512P}$ mice showed the cleft palate.** (**a**) *Atp6v0a1*$^{+/+}$ and *Atp6v0a1*$^{A512P/A512P}$ pups at postnatal day 1 (P1). White arrows indicate the location of stomach. Note that the absence of milk in the stomach of the *Atp6v0a1*$^{A512P/A512P}$ pups. (**b**) Cleft palate in *Atp6v0a1*$^{A512P/A512P}$ pups (arrowheads). Scale bars, 1 mm.

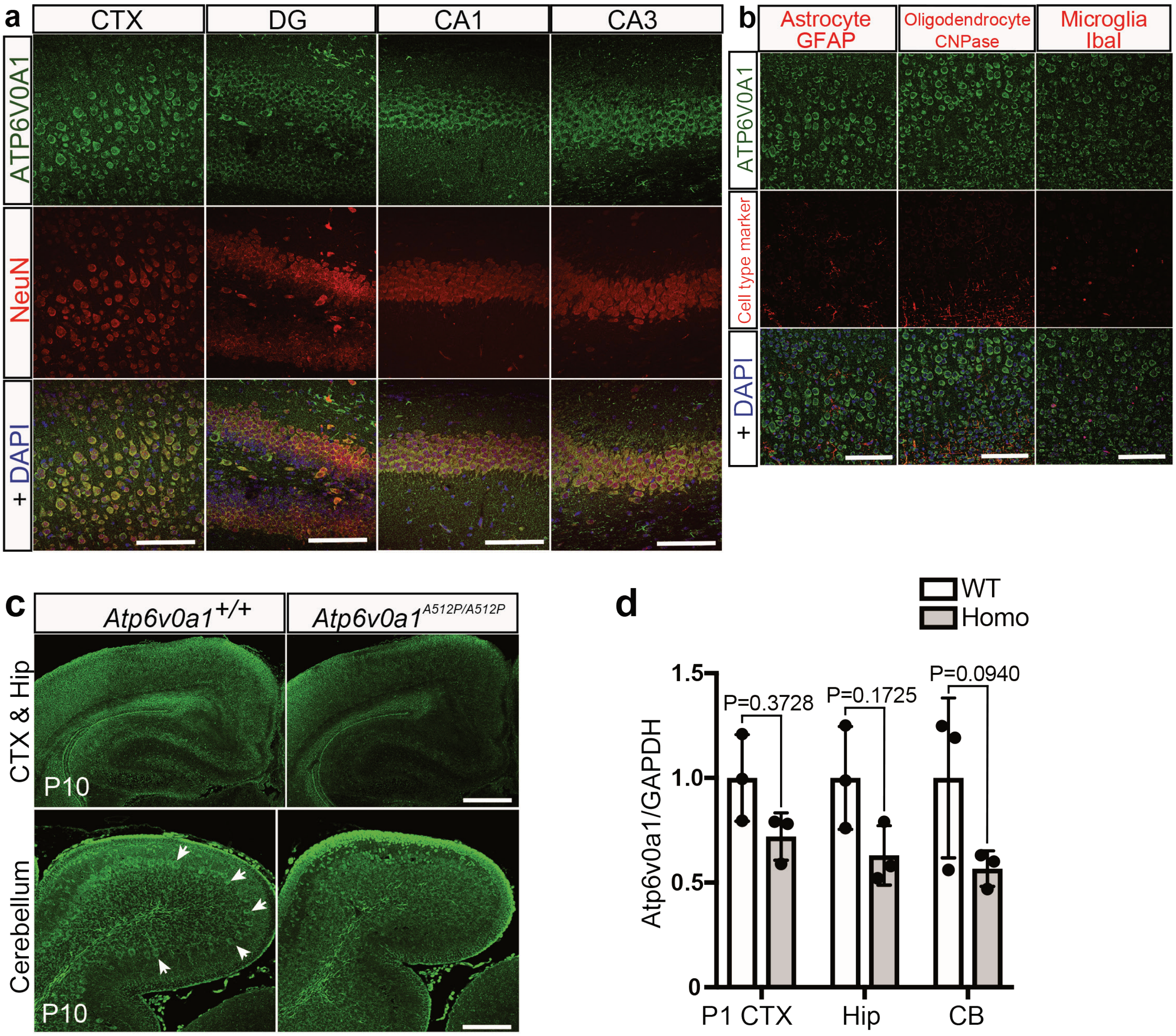

Supplementary Fig. 6 Atp6v0a1 protein expression in the cortex, the hippocampus and the cerebellum. (**a**) Immunostaining of Atp6v0a1 (green) and a neuronal marker NeuN (red) in the cortex (CTX), hippocampal dentate gyrus (DG), CA1 and CA3 regions of the hippocampus in adult wild-type (WT) mice. Most Atp6v0a1 proteins were present in NeuN-positive neurons. (**b**) Immunostaining of Atp6v0a1 (green), an astrocyte marker GFAP (red), a oligodendrocyte marker CNPase (red) and a microglia marker Iba1 (red) in the cortal layer VI/V of adult WT mice. Very few Atp6v0a1 were colocalized with these markers. (**c**) Atp6v0a1 distribution in CTX and the hippocampus (Hip, coronal sections) and in the cerebellum (sagittal sections) of *Atp6v0a1*$^{+/+}$ (WT) and *Atp6v0a1*$^{A512P/A512P}$ (Homo) pups at P10. The protein level of Atp6v0a1 appears to be decreased in the neurons of CTX and Hip and in Purkinje cells (arrowheads) of the cerebellum in the *Atp6v0a1*$^{A512P/A512P}$ brain. We consider that marginal staining of the cerebellum in *Atp6v0a1*$^{A512P/A512P}$ brain is nonspecific. (**d**) Quantification of Atp6v0a1 protein level at P1. Results of three independent experiments showed a tendency toward reduced Atp6v0a1 protein levels in all three regions of

Atp6v0a1$^{A512P/A512P}$ brains, though differences did not reach statistical significance. Two way ANOVA with Sidak's multiple comparisons test (**d**) was used for comparing WT with *Atp6v0a1*$^{A512P/A512P}$. Data represented as mean values ± standard deviation (**d**). Scale bars, 100 μm. Data in **a**, **b** and **c** are representative of more than twice independent experiments. Source data are provided as a Source Data file.

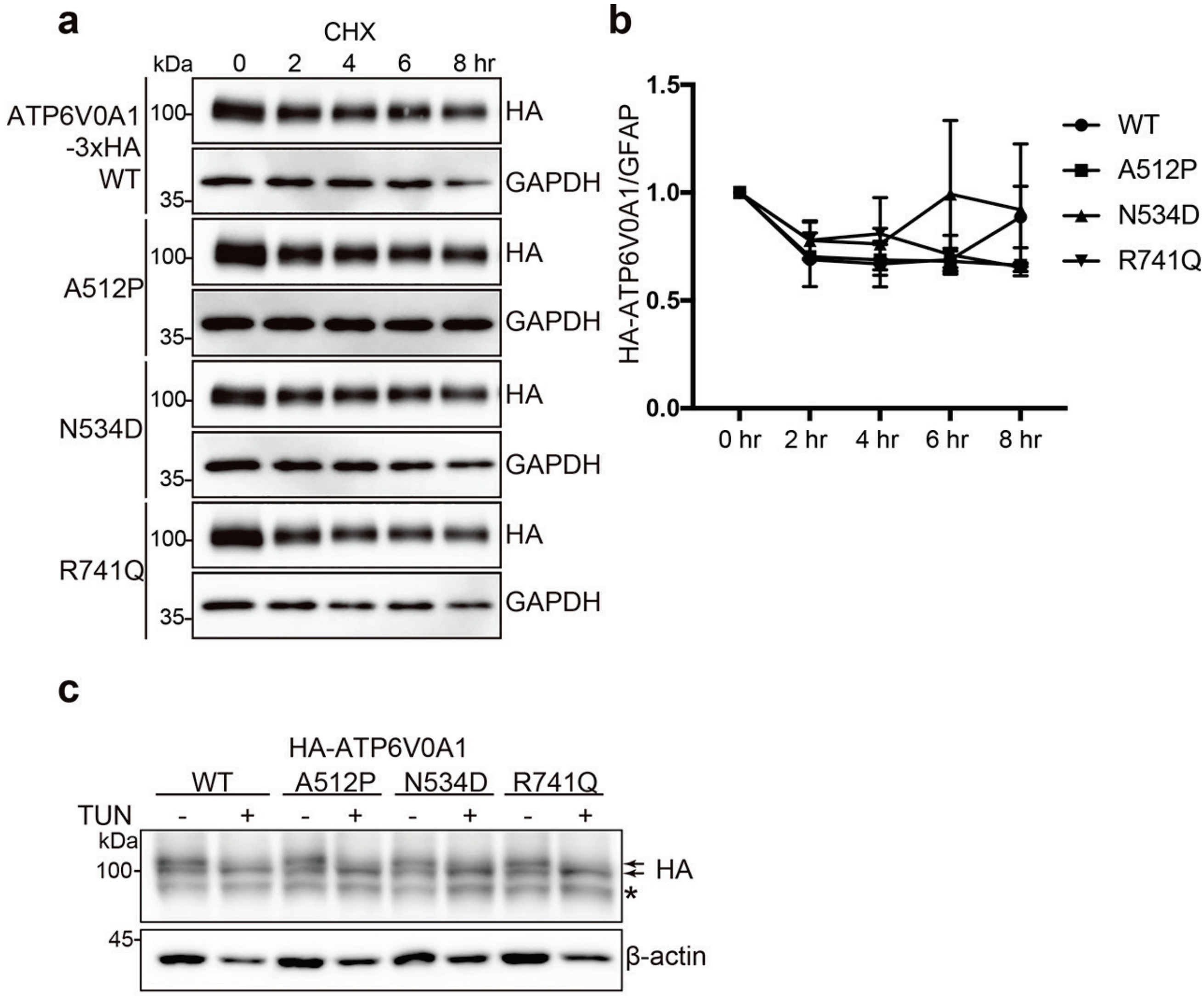

Supplementary Fig. 7 Protein stability and glycosylation of ATP6V0A1^{WT} and three mutants. (**a**) Immunoblot analysis of HA and GAPDH loading control in the ATP6V0A1-3xHA expressing HEK293FT stable cell lines treated with cycloheximide (CHX) for 0-8 h. (**b**) Quantification of the band intensity of the immunoblots of three independent experiments, which shows no difference among wild-type and mutant ATP6V0A1-3xHA. Data represented as mean values ± standard deviation. (**c**) Immunoblot analysis of HA and β-actin loading control in the HA-ATP6V0A1 expressing N2A stable cell lines treated with Tunicamycin (TUN) for 24 h. Untreated HA-ATP6V0A1 cells showed immature 100 kDa band and glycosylated 116 kDa band (arrows), while TUN treated cells showed only 100 kDa band. Asterisk (*) shows the non-specific band. Experiments were repeated three times. Data in **a** and **c** are representative of three independent experiments. Source data are provided as a Source Data file.

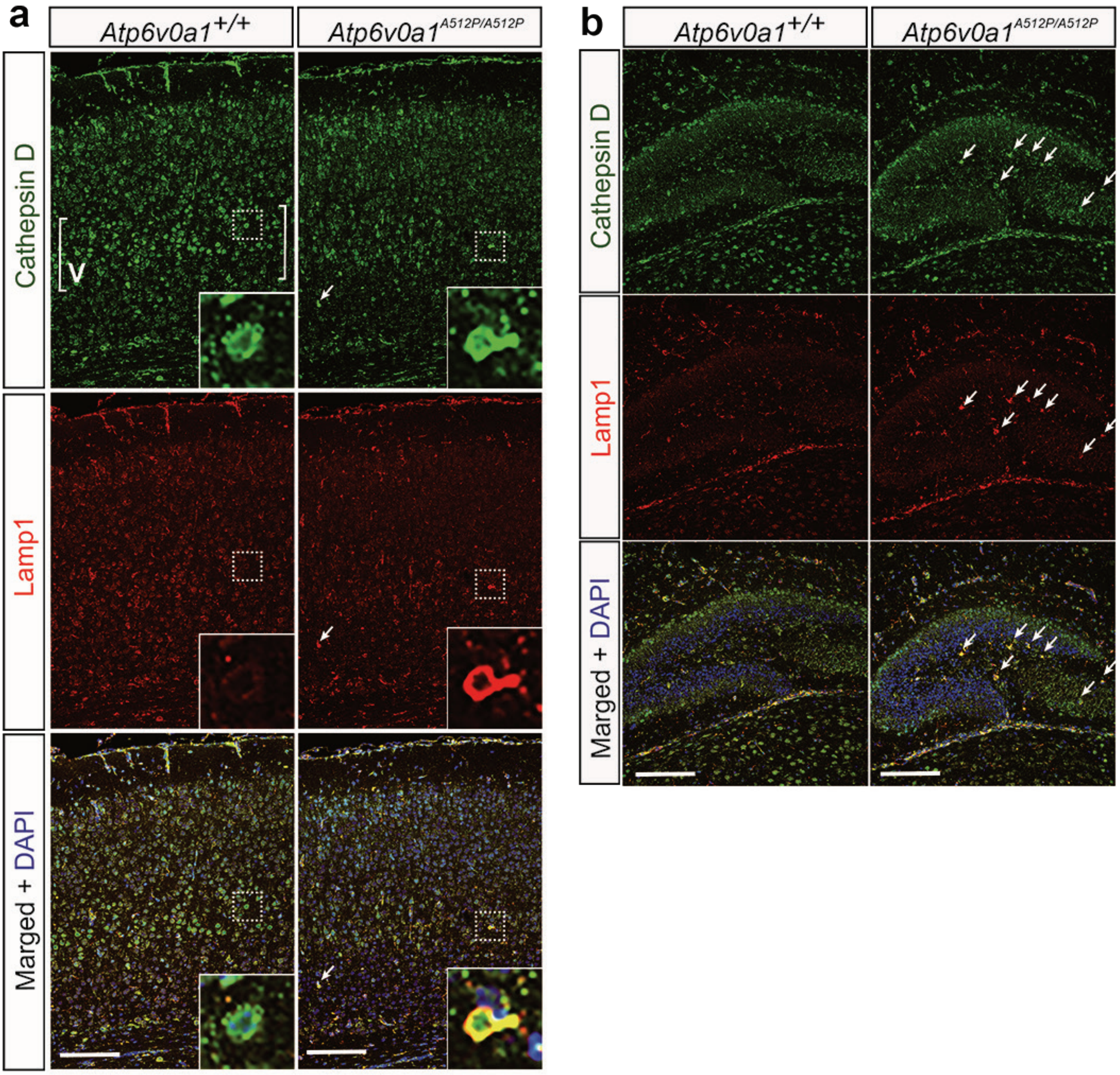

Supplementary Fig. 8 Distribution of Cathepsin D and Lamp1 proteins in the cortex and hippocampal dentate gyrus of *Atp6v0a1*$^{+/+}$ and *Atp6v0a1*$^{A512P/A512P}$ pups. (**a**, **b**) Immunostaining of the lysosomal enzyme cathepsin D (green) and the lysosomal membrane marker Lamp1 (red) in the cortex (**a**) and hippocampal dentate gyrus (**b**) of *Atp6v0a1*$^{+/+}$ and *Atp6v0a1*$^{A512P/A512P}$ pups at P10. Enlarged images of the boxed areas in layer V of the cortex and arrows in *Atp6v0a1*$^{A512P/A512P}$ indicate expanded distributions of cathepsin D and Lamp2 proteins. Data in **a** and **b** are representative of three independent experiments. Scale bars, 100 µm.

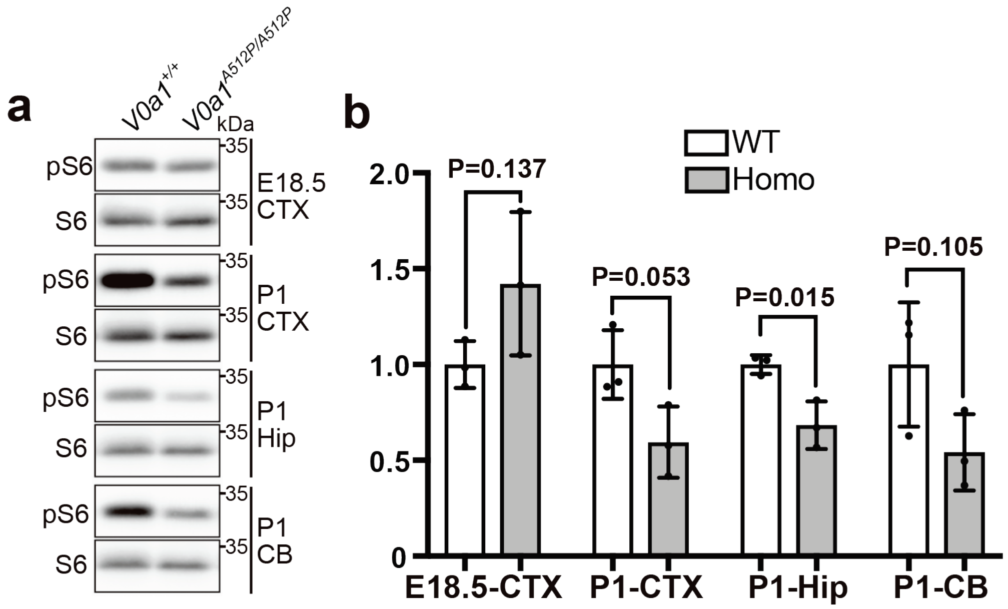

Supplementary Fig. 9 Phospho-S6 (pS6) protein level in *Atp6v0a1*$^{+/+}$ and *Atp6v0a1*$^{A512P/A512P}$ pups at early developmental stage. (**a**) Immunoblot analysis of pS6 and total S6 in the cortex (CTX), hippocampus (Hip) and cerebellum (CB) of *Atp6v0a1*$^{+/+}$ (WT) and *Atp6v0a1*$^{A512P/A512P}$ (Homo) pups at embryonic day 18.5 (E18.5) and P1. (**b**) The pS6 intensity was normalized to the total S6 intensity and ratios to average intensity of WT pups were shown. Reduced pS6 signals in the hippocampus of P1 pups were recognized. Data represented as mean values ± standard deviation of three independent experiments. Two way ANOVA with Sidak's multiple comparisons test was used for comparing WT with *Atp6v0a1*$^{A512P/A512P}$ (**b**). Data in **a** are representative three independent experiments. Source data are provided as a Source Data file.

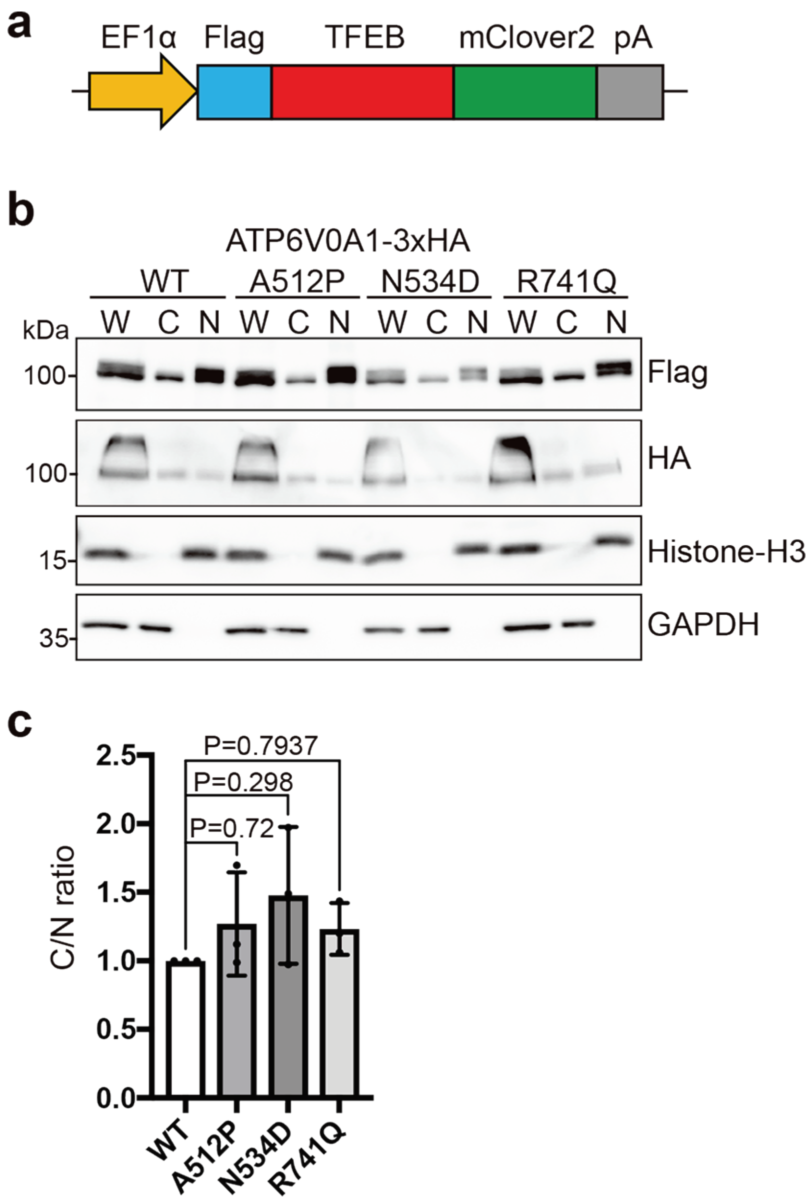

Supplementary Fig. 10 Cytoplasmic and nuclear localization of Flag-TFEB-mClover2 in ATP6V0A1-3xHA stable cell line. (**a**) Expression construct of N terminal Flag and C terminal mClover2 tagged TFEB driven by the EF1α promoter and SV40 polyA terminal (pA). (**b**) Immunoblot analysis of Flag, HA, Histone-H3 and GAPDH loading control in the cellular fraction of ATP6V0A1-3xHA expressing HEK293FT stable cell lines treated after Flga-TFEB-mClover2 transfection for 24 h. Whole cell lysate (W), cytosolic (C) and nuclear (N) fraction. (**c**) Quantification of C/N ratio of the immunoblots of three independent experiments, which shows no difference among wild-type and mutant expressing cells. Two way ANOVA with Sidak's multiple comparisons test was used for comparing among ATP6V0A1 mutants. Data represented as mean values ± standard deviation. Data in **b** are representative of three independent experiments. Source data are provided as a Source Data file.

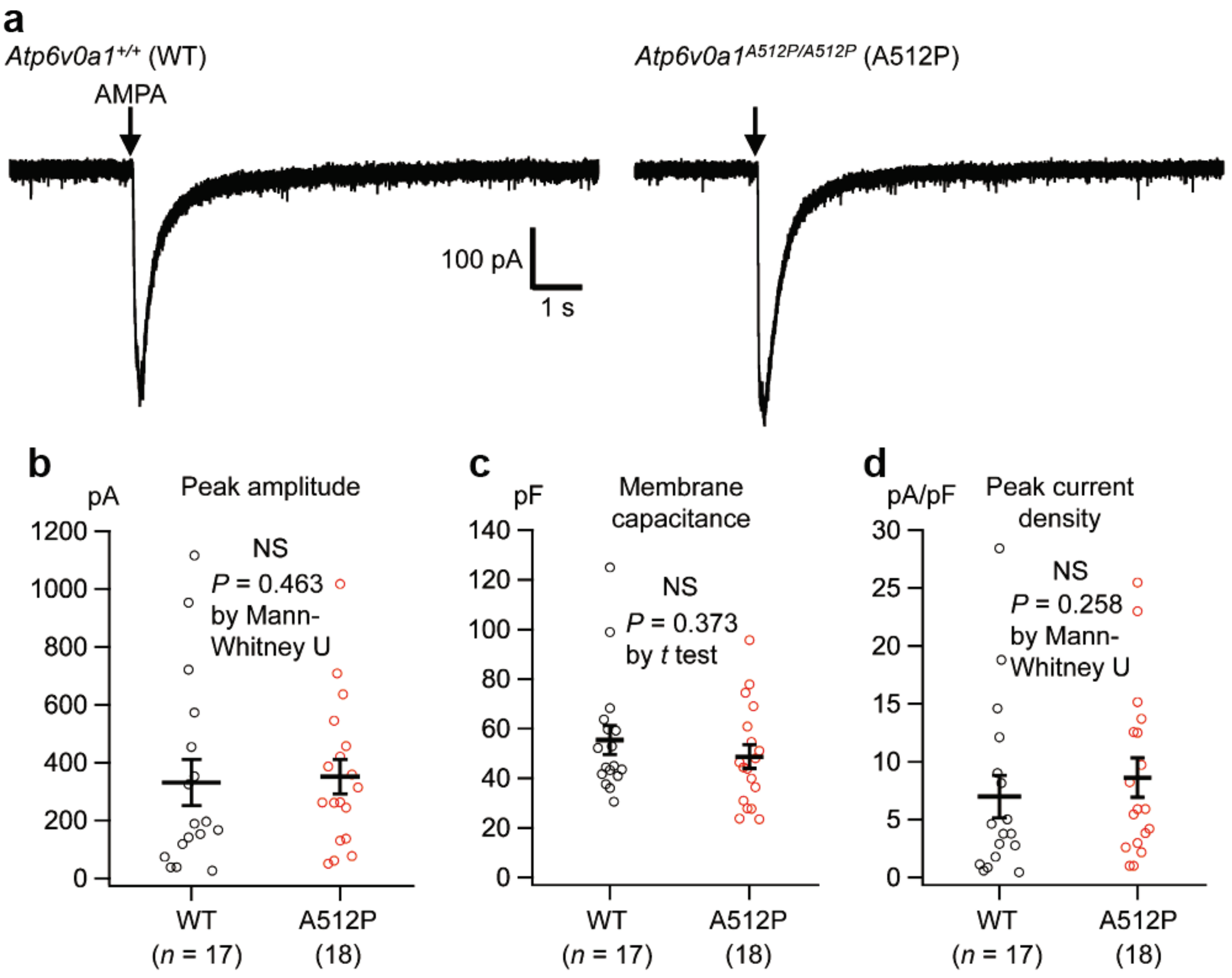

Supplementary Fig. 11 Comparison of AMPA-induced currents in *Atp6v0a1*$^{+/+}$ (WT) and *Atp6v0a1*$^{A512P/A512P}$ (A512P) neurons. (A) Representative traces of inward membrane currents induced by puff application of 100 μM AMPA in WT and A512P neurons. Neurons were voltage-clamped at −70 mV. (B) The peak amplitude of the average of 5 current responses in each neuron was plotted and compared between WT and A512P neurons. There were no significant differences in the amplitude (NS, P = 0.463 by Mann-Whitney U test). (C) The membrane capacitance of recorded neurons was similar between WT and A512P (P = 0.373 by two-sided t test). (D) The peak current density, calculated by dividing the amplitude by the membrane capacitance of the neuron, was also similar (P = 0.258 by Mann-Whitney U test), indicating similar postsynaptic densities of AMPA receptors between WT and A512P neurons. Horizontal and error bars indicate mean ± s.e.m.

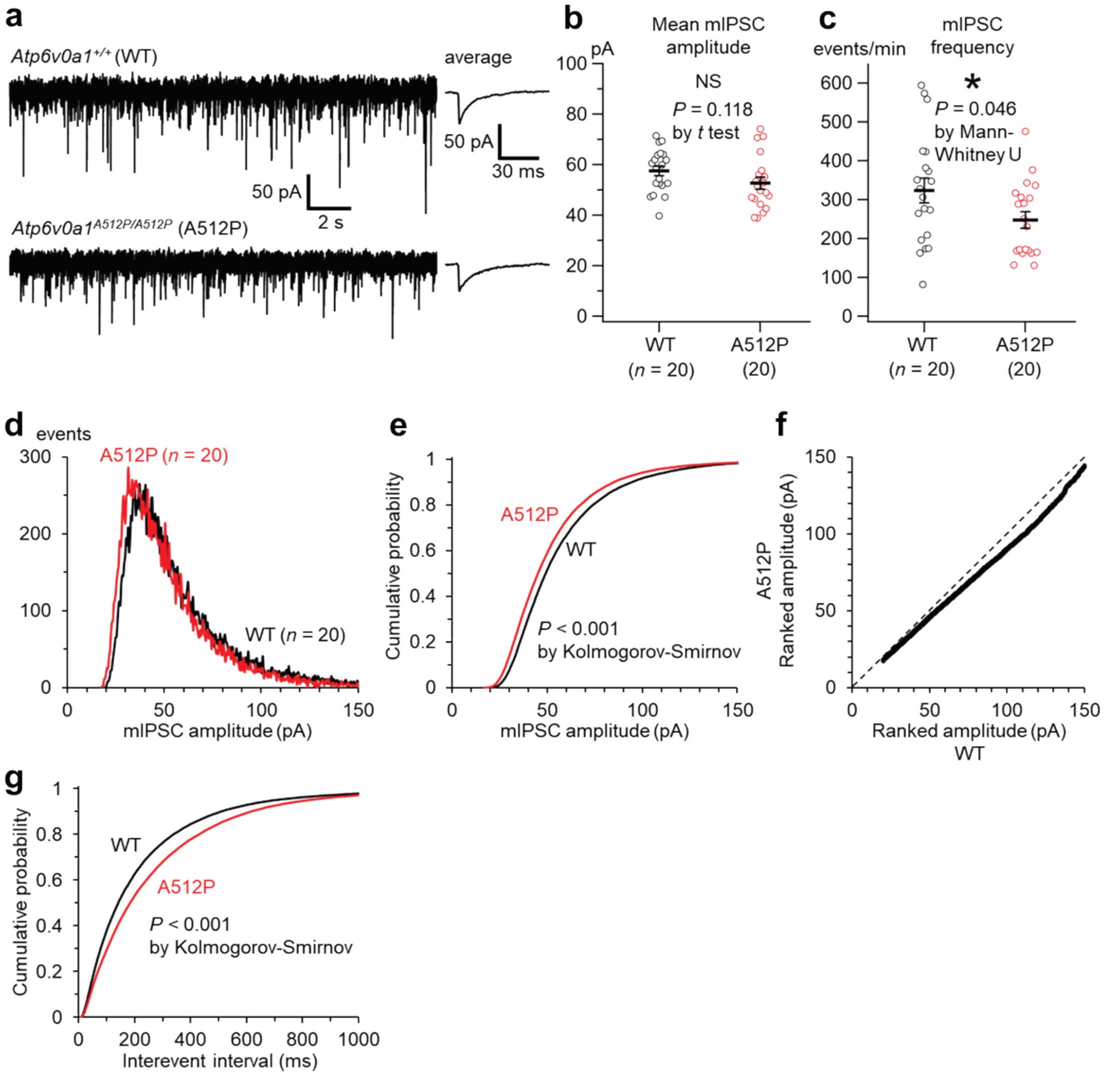

Supplementary Fig. 12 Comparison of mIPSCs in *Atp6v0a1*$^{+/+}$ (WT) and *Atp6v0a1*$^{A512P/A512P}$ (A512P) neurons. (**a**) Representative traces of mIPSCs in WT and A512P neurons. The average of individual mIPSCs in each trace is shown on the right. Note that the time course of single mIPSCs was longer than that of mEPSCs. (**b**, **c**) The mean amplitude (**b**) and frequency (**c**) of 1,000 consecutive mIPSCs in each neuron are plotted and compared. The mean amplitude in A512P neurons was not significantly different (NS, $P = 0.118$ by two-sided t test) from that in WT neurons, whereas the frequency in A512P neurons was significantly lower than that in WT neurons ($P = 0.046$ by Mann-Whitney U test). Horizontal and error bars indicate mean ± s.e.m. (**d**, **e**) Histograms (**d**) and cumulative distributions (**e**) of the amplitudes of 20,000 events of mIPSCs in the WT and A512P neuron groups. The bin widths were 0.5 pA in (**d**) and 0.1 pA in (**e**). The plots indicated a significant leftward shift of the distribution in the A512P group compared with that in the WT group ($P < 0.001$ by Kolmogorov-Smirnov test). (**f**) Ranked mIPSC amplitudes in ascending order in the A512P group were plotted against those in the WT group. The dashed line indicates the level when the amplitude

distributions are the same. The slope of the plots was lower than the dashed line because of the smaller proportion of large mIPSCs in the A512P group. (**g**) Cumulative distributions of interevent intervals of mIPSCs, indicating a significantly larger proportion of long intervals in the A512P group ($P < 0.001$ by Kolmogorov-Smirnov test).

Supplementary Table 1 Prediction of the pathogenicity of *ATP6V0A1* (NM_001130020.1) variants

Individual	cDNA change	Amino acid change	GnomAD	SIFT	Polyphen2 HumVar	Mutation Taster	CADD Phred	MCAP
1&2	c.2222G>A	p.(Arg741Gln)	-	0	Probably damaging 1.000	Disease causing 0.999	33	Possibly Pathogenic 0.596
3	c.1534G>C	p.(Ala512Pro)	-	0.184	Benign 0.218	Disease causing 0.999	17.12	Possibly Pathogenic 0.048
4	c.1600A>G	p.(Asn534Asp)	2 of 251,286 alleles	0.001	Probably damaging 0.914	Disease causing 0.999	29	Possibly Pathogenic 0.436

Individual	cDNA change	Amino acid change	GnomAD	NetGene2	BDGP	HSF3.0
4	c.196+1G>A	Not examined	2 of 251,356 alleles	Confidence 0.71 →disappeared	Score 0.45 →disappeared	Score 73.45 →46.62 Site broken

N/A = not applicable.

GnomAD (http://gnomad.broadinstitute.org/)

SIFT (http://sift.jcvi.org/): scores < 0.05 indicate that substitutions are predicted to be intolerant.

PolyPhen-2 (http://genetics.bwh.harvard.edu/pph2/): scores are evaluated as 0.000 (most probably benign) to 0.999 (most probably damaging).

MutationTaster (http://www.mutationtaster.org/): rapid evaluation of DNA sequence alterations. The alterations are classified as

disease-causing or polymorphisms. Probability value is shown.
CADD (http://cadd.gs.washington.edu/score): PHRED scores of 10–20 and > 20 are regarded as deleterious and the 1% most deleterious, respectively.
M-CAP (http://bejerano.stanford.edu/mcap/): it correctly dismisses 60% of rare, missense variants of uncertain significance in a typical genome at 95% sensitivity. Scores of > 0.025 are regarded as possibly pathogenic.
NetGene2 (http://www.cbs.dtu.dk/services/NetGene2/), BDGP (http://www.fruitfly.org/), HSF3.0 (http://www.umd.be/HSF3/)

Supplementary Table 2. Summary of clinical features of individuals with *ATP6V0A1* variants

Feature	Individual 1	Individual 2	Individual 3	Individual 4
Gender	Female	Male	Male	Male
Current age	24 yr	15 yr	7 yr	8 yr
Clinical diagnosis	Unclassified DEE	West syndrome, then unclassified DEE	Early myoclonic encephalopathy	Multifocal epilepsy and global developmental delay
Ethnicity	Japanese	Japanese	Japanese	Islaeri
Consanguinity	No	No	No	No
Variant	*de novo*	*de novo (likely)*	*recessive*	*recessive*
Abnormalities during pregnancy	Coiling of the unmbilical cord	In vitro fertilazation, vacuum delivery due to weak labor pain and arrest of labor	Polyhydramnios at 26 weeks of gestation	In Vitro Fertilization, Maternal Hypertention
Gestational age in weeks	40	36	36	40
Fetal distress	No	Not fully informed. 1 day in a incubator with phototherapy	No	No
Birth weight (SD), length (SD), HC (SD)	2956 g (-0.1), 47.0 cm (-1.3), 31.5 cm (-1.5)	2150 g (-1.0), 43.5 cm (-1.2), 30.5 cm (-1.0)	2620 g (-0.1), 46 cm (-0.4), 33.5 cm (+0.7)	3550 g (65.9 percentile), length unknown, 35 cm (66.4 percentile)
Initial symptom	Developmental delay showing head control at 5 mo	Developmental delay showing no eye contact at 5 mo	Salivation, poor feeding and obstructive apneic attacks soon after birth	CPS (partial seizure with impaired consciousness) at 5 weeks
Seizure onset	7 mo	6 mo	27 days	5 weeks
Type of first seizure	Staring with oral and arm automatism	Epileptic spasms in cluster	Subtle seizures, such as pedaling and rowing	Staring with clonic movements of lower limbs
Type of other seizure	Clonic, FIAS, GTC	Tonic, GTC, FIAS	Epileptic spasms at 29 days, tonic sz of upper extremities with staring, erratic myoclonus	Tonic, GTC at 1.3 years
EEG findings at the onset	Focal sharp waves at left midtemporal and central area	Hypsarrhythmia with prominent periodicity	Suppression burst (multifocal epileptic discharges predominantly at left occipitotemporal area)	Electroclinical seizure with fast frontal activity
EEG findings during the course	Multifocal spikes or polyspikes at 2 yr 10 mo, then no epileptic discharges temporarily. Infrequent multifocal spike-and-slow waves at 9 years and 18 years	No epileptic discharges at 7 years	No epileptic discharges at 62 days. Low-amplitude suppression burst pattern at 8 mo, then almost flat EEG at 10 mo and 3 years	High-amplitude spikes at bilateral frontocentral area with no activation during sleep
Response to AEDs	Refractory to VPA, CZP, CBZ, PRM. Temporarily effective: PB, PLP, PHT, ZNS. Partially effective: LEV, LTG	Refractory to Vit.B6, VPA. Spasms disappeared with ACTH. GTC disappeared with ZNS. LEV was temporarily effective for FIAS. PER and TPM were inffective.	Partially effective: PB, ZNS, CZP, LEV, KBr Refractory to VPA, CLB	Controlled by pyridoxine for few weeks. Refractory to phenobarbital, levotiracetam and topiramate. Partially effective: oxcarbazepine and valproic acid
Course of epilepsy	Intractable: GTC, FIAS	Intractable: FIAS, monthly	Intractable: erratic myoclonus during awake state	Intractable: Tonic seizres
Development	Sitting alone at 11 mo, standing alone at 17 mo, walking alone at 22 mo, meaningful words at 3 yr 6 mo	Sitting alone at 14 mo, standing alone at 4 yr, walking alone at 5 yr	No head control, no eye contact, no social smile	At the age of 18 months walked around furnitures and said 10 words. walking alone at 26 mo after oxcarbazepine tretment cessation.
Intellectual disability	Yes, profound	Yes, profound. No meaningful words	Yes, profound	Significant global difficalties: Motor, Language, learning dificulties
DQ/IQ	12 at 6 yr, 7 at 8 yr	10 at 14 yr	<10 at 7 yr	79 at 34 mo
Other neurological findings	Hand stereotypy	Autism, hyperactivity, irritability, self-mutilation	Bedridden with spastic quadriplegia and systemic joint contractures	Attention deficit disorder of childhood
Dysmorphisms	None	None	Overlapping fingers	Mild
Cleft palate	None	None	None	None
Others		Dizygotic twin. A female sib showed normal development and no seizure.	Intubation at 9 days, tracheostomy at 2 mo, gastrostomy and fundoplication at 6 mo, and laryngotracheal separation at 8 mo. Osteoporosis with bone fracture, tendency for diarrhea	Abnormal thyroid tests with no need of treatment (high TSH with normal fT4)
Current body weight (SD), length (SD), HC (SD)	24.65 kg (-0.88), 120.2 cm (-2.0), 48.0 cm (-2.7) at 9 yr 4 mo	39.0 kg (-1.8), 157.0cm (-1.7), unknown at 15 yr 2 mo	10.7 kg (-2.2), 77.1 cm (-5.2), unknown at 3 yr 4 mo	Unknown
Brain MRI	Normal in infancy	Enlarged lateral and 3rd ventricles at 6 mo and 4 yr	Enlarged lateral venticles and hypoplasia of the cerebellar vermis at 10 days. Progressively severe brain atrophy at 6 mo and 3 yr	Normal in infancy. Mild cerebellar atrophy on repeated brain MRI at age 3 years. Severe atrophy of brainstem and cerbellum at age 6 years.

AEDs, antiepileptic drugs; BGA, background activity; CBZ, carbamazepine; CLB, clobazam; CSF, cerebrospinal fluid; CZP, clonazepam; EEG, electroencephalogram; FIAS, focal impaired awareness seizure; GM, gross movement; GTC, generalized tonic convulsion; HC, head circumference; LEV, levetiracetam; mo, month(s); LTG, lamotrigine; MRI, magnetic resonance imaging; NA, not available; NR, no response; PB, phenobarbital; PER, perampanel; PHT, phenytoin; PLP, pyridoxal phosphate; PN, pyridoxine; S-B, suppression-burst; SD, standard deviation; SIA, seizures with impaired awareness; SW, spike and slow wave; sz, seizure(s); TPM, topiramate; VPA, valproic acid; wk, week(s); yr, year(s).
