# Supplementary material for: ATP6V0A1 encoding the a1-subunit of the V0 domain of vacuolar H+-ATPases is essential for brain development in humans and mice
Source: Nat Commun. 2021 Apr 8;12:2107. doi: 10.1038/s41467-021-22389-5 (PMC8032687; doi:10.1038/s41467-021-22389-5)
Supplement: Supplementary file 3 — Description of Additional Supplementary Files [file 41467_2021_22389_MOESM3_ESM.pdf]

Description of Additional Supplementary Files

File Name: Supplementary Movie 1
Description: The ataxia of a long-surviving Atp6v0a1A512P/A512P pup at postnatal day 9.
