# Supplementary material for: ATP6V0A1 encoding the a1-subunit of the V0 domain of vacuolar H+-ATPases is essential for brain development in humans and mice
Source: Nat Commun. 2021 Apr 8;12:2107. doi: 10.1038/s41467-021-22389-5 (PMC8032687; doi:10.1038/s41467-021-22389-5)
Supplement: Supplementary file 5 — Reporting Summary [file 41467_2021_22389_MOESM5_ESM.pdf]

nature research

Corresponding author(s): Hirotomo Saitsu, Naomichi Matsumoto

Last updated by author(s): July 20, 2020

Reporting Summary

Nature Research wishes to improve the reproducibility of the work that we publish. This form provides structure for consistency and transparency in reporting. For further information on Nature Research policies, see Authors & Referees and the Editorial Policy Checklist .

Statistics

For all statistical analyses, confirm that the following items are present in the figure legend, table legend, main text, or Methods section.

n/a	Confirmed	
☐	☒	The exact sample size (*n*) for each experimental group/condition, given as a discrete number and unit of measurement
☐	☒	A statement on whether measurements were taken from distinct samples or whether the same sample was measured repeatedly
☐	☒	The statistical test(s) used AND whether they are one- or two-sided<br>*Only common tests should be described solely by name; describe more complex techniques in the Methods section.*
☐	☒	A description of all covariates tested
☐	☒	A description of any assumptions or corrections, such as tests of normality and adjustment for multiple comparisons
☐	☒	A full description of the statistical parameters including central tendency (e.g. means) or other basic estimates (e.g. regression coefficient) AND variation (e.g. standard deviation) or associated estimates of uncertainty (e.g. confidence intervals)
☐	☒	For null hypothesis testing, the test statistic (e.g. *F*, *t*, *r*) with confidence intervals, effect sizes, degrees of freedom and *P* value noted<br>*Give P values as exact values whenever suitable.*
☒	☐	For Bayesian analysis, information on the choice of priors and Markov chain Monte Carlo settings
☒	☐	For hierarchical and complex designs, identification of the appropriate level for tests and full reporting of outcomes
☐	☒	Estimates of effect sizes (e.g. Cohen's *d*, Pearson's *r*), indicating how they were calculated

Our web collection on statistics for biologists contains articles on many of the points above.

Software and code

Policy information about availability of computer code

Data collection

Human genome sequence was performed by Illumina HiSeq 2500 with 101 bp paired-end reads. Genome information of mouse Atp6v0a1 was taken form UCSC Genome Browser. High resolution melting (HRM) of Atp6v0a1 mutant mice was analyzed using an Eco 48 Real Time PCR system (PCRmax, Stone, UK).

Data analysis

Copy number alterations were analyzed using the Chromosome Analysis Suite (ChAS; Affymetrix). Cell count analysis, immunoblotting band analysis and TUNEL analysis were performed using the Image J software. Statistical analyses except for electrophysiological data were carried out using GraphPad Prism7. For electrophysiological data, statistical analysis was carried out using IBM SPSS ver.25 software.

Following softwares and databases were used for whole exome sequence analysis.
NovoAlign v3.02 (http://www.novocraft.com/products/novoalign/)
Picard v1.55 (https://broadinstitute.github.io/picard/)
GATK v2.7 (https://software.broadinstitute.org/gatk/)
Annovar (http://annovar.openbioinformatics.org/en/latest/)
gnomAD v2.1.1(https://gnomad.broadinstitute.org/)
SIFT (http://sift.jcvi.org/)
PolyPhen-2 (http://genetics.bwh.harvard.edu/pph2/)
MutationTaster (http://www.mutationtaster.org/)
CADD v1.3 (https://cadd.gs.washington.edu/)
M-CAP v1.3 (http://bejerano.stanford.edu/mcap/)

For manuscripts utilizing custom algorithms or software that are central to the research but not yet described in published literature, software must be made available to editors/reviewers. We strongly encourage code deposition in a community repository (e.g. GitHub). See the Nature Research guidelines for submitting code & software for further information.

Data

Policy information about availability of data

All manuscripts must include a data availability statement. This statement should provide the following information, where applicable:

- Accession codes, unique identifiers, or web links for publicly available datasets
- A list of figures that have associated raw data
- A description of any restrictions on data availability

Exome data are available in the Human Genetic Variation Database [http://www.hgvd.genome.med.kyoto-u.ac.jp/repository/HGV0000012.html]. Raw sequence and individual-level genotype data can be provided via formal collaboration because of the contents of the obtained informed consent. Primary data of interest is provided in the source data file of this paper. All the other data are available within the article and its Supplementary Information Files from the corresponding author upon reasonable request. Data from RefSeq and Uniprot database are following: NM_001130020.1 (https://www.ncbi.nlm.nih.gov/nuccore/NM_001130020.1), NM_001167827.3 (https://www.ncbi.nlm.nih.gov/nuccore/NM_001167827.3), NP_058616.1 (https://www.ncbi.nlm.nih.gov/protein/NP_058616.1), NP_001123492.1 (https://www.ncbi.nlm.nih.gov/protein/NP_001123492.1), NP_058616.1 (https://www.ncbi.nlm.nih.gov/protein/NP_058616.1), NP_990055.1 (https://www.ncbi.nlm.nih.gov/protein/NP_990055.1), NP_997837.1 (https://www.ncbi.nlm.nih.gov/protein/NP_997837.1), NP_651672.1 (https://www.ncbi.nlm.nih.gov/protein/NP_651672.1), NP_014913.3 (https://www.ncbi.nlm.nih.gov/protein/NP_014913.3), and Q93050 (https://www.uniprot.org/uniprot/Q93050).

Field-specific reporting

Please select the one below that is the best fit for your research. If you are not sure, read the appropriate sections before making your selection.

[x] Life sciences [ ] Behavioural & social sciences [ ] Ecological, evolutionary & environmental sciences

For a reference copy of the document with all sections, see nature.com/documents/nr-reporting-summary-flat.pdf

Life sciences study design

All studies must disclose on these points even when the disclosure is negative.

Sample size	We identified four individuals with ATP6V0A1 variants in 700 individuals with developmental and epileptic encephalopathies (three males and a female).<br>The fluorescence intensity of five taken images in each neuro2a cell lines was taken. The immunoblotting band intensity, immnohistochemical samples and TUNEL samples from three WT and Atp6v0a1A512P/A512P pups brain were taken. No statical estimation of sample size was performed. These numbers are comparable to those used in previous publications, and ensure reproducibility.
Data exclusions	No data were excluded from the analysis.
Replication	All experiments were repeated 2 or more times. All significant changes were reproducible between experimental repeats.
Randomization	For mouse analysis, we are comparing phenotypes between WT and Atp6v0a1A512P/A512P pups. Cells and sections used for imaging were selected randomly.
Blinding	Blinding was not possible due to clear/visible phenotypic diffrerences.

Reporting for specific materials, systems and methods

We require information from authors about some types of materials, experimental systems and methods used in many studies. Here, indicate whether each material, system or method listed is relevant to your study. If you are not sure if a list item applies to your research, read the appropriate section before selecting a response.

Materials & experimental systems

n/a	Involved in the study	
[ ]	[x]	Antibodies
[ ]	[x]	Eukaryotic cell lines
[x]	[ ]	Palaeontology
[ ]	[x]	Animals and other organisms
[ ]	[x]	Human research participants
[x]	[ ]	Clinical data

Methods

n/a	Involved in the study	
[x]	[ ]	ChIP-seq
[x]	[ ]	Flow cytometry
[ ]	[x]	MRI-based neuroimaging

Antibodies

Antibodies used	rabbit anti-ATP6V0A1 (gift from Dr. Sato), rabbit anti-ATP6V1A (ab137574, Abcam), mouse anti-β-actin (MA1-140, Thermo Fisher Scientific), mouse anti-calbindin D-28k (300, Swant), rabbit anti-cathepsin D (ab75852, Abcam), goat anti-cathepsin D (sc-6489, Santa Cruz), mouse anti-CNPase (ab6319, Abcam), mouse anti-Flag (2H8) (KO602, Transgenic inc.), mouse anti-GAPDH (60004-1-Ig, Proteintech), mouse anti-GFAP (G3893, Sigma-Aldrich), rat anti-HA-Tag (3F10) (11867423001, Roche), rabbit Histone-H3 (17168-AP, Proteintech), goat anti-IbaI (ab5076, Abcam), rat anti-LAMP1 (1D4B, DSHB), mouse anti-LAMP-2 (ABL-93-s, DSHB), mouse anti-NeuN (MAB377, Milipore), rabbit anti-LC3 (PM036, MBL), rabbit phosphor-S6 (Ser235/236) (#4856, Cell Signaling), rabbit anti-mTor (#2972, Cell Signaling Technology), rabbit phospho-mTor (Ser2448) (#5536, Cell Signaling Technology), mouse anti-PSD95 (7E3-1B8) (MA1-046, Thermo Fisher Scientific), rabbit anti-S6 ribosomal protein (#2217, Cell Signaling Technology), guinea pig anti-vGluT1 (AB5905, Millipore), Alexa Fluor 488 or 546 Donkey anti-mouse IgG, anti-rat IgG and anti-goat IgG (Thermo Fisher Scientific), goat anti-rabbit and anti-mouse antibodies conjugated with horseradish peroxidase (Jackson ImmunoResearch, West Grove, PA).
Validation	The antibodies were validated in the manufacture's website (Abcam, Thermofisher, Swant, Santa Cruz, Transgenic inc., Proteintech, Sigma-Aldrich, Roche, DSHB, Millipore, MBL, Cell signaling technology, Jackson lab) and the following study: Sato SB. Interference with the endosomal acidification by a monoclonal antibody directed toward the 116 (100)-kD subunit of the vacuolar type proton pump. The Journal of Cell Biology 127, 39-53 (1994).

Eukaryotic cell lines

Policy information about cell lines

Cell line source(s)	Neuro2a cell line was derived from Cell Biology collection, ATCC. HEK293FT cell line was derived from Thermo Fisher Scientific.
Authentication	Commercially obtained cell lines, neuro2a and HEK293FT were authenticated by ATCC or Thermo Fisher.
Mycoplasma contamination	Neuro2a cell line and HEK293FT cell line were not tested, but we could not detect mycoplasma in DAPI staining.
Commonly misidentified lines (See ICLAC register)	*Name any commonly misidentified cell lines used in the study and provide a rationale for their use.*

Animals and other organisms

Policy information about studies involving animals; ARRIVE guidelines recommended for reporting animal research

Laboratory animals	Male and female ICR mice between 8-12 weeks were used in this study and maintained in the Animal facility of Hamamatsu university.
Wild animals	This study did not involve wild animals.
Field-collected samples	This study did not involve field-collected samples.
Ethics oversight	All animal experiments were performed in accordance with the guidelines of the Physiological Society of Japan and approved by the Institutional Animal Care and Use Committee of the Hamamatsu University School of Medicine.

Note that full information on the approval of the study protocol must also be provided in the manuscript.

Human research participants

Policy information about studies involving human research participants

Population characteristics	We identified four individuals with ATP6V0A1 variants in 700 individuals with developmental and epileptic encephalopathies (three males and a female), and described clinical features of these four individuals as well as functional analysis of identified missense variants. Therefore, we are unable to delineate possible full clinical features caused by ATP6V0A1 variants as we focused on roles of ATP6V0A1 in developmental and epileptic encephalopathies. Because ATP6V0A1 is an autosomal gene, gender of four individuals may not affect clinical features caused by ATP6V0A1 variants
Recruitment	We found three Japanese and an Israeli individuals with developmental and epileptic encephalopathies with ATP6V0A1 variants. This nationality bias might affect clinical features of ATP6V0A1 variants described in this study.
Ethics oversight	Experimental protocols were approved by the institutional review boards of Yokohama City University, Hamamatsu University and Showa University School of Medicine.

Note that full information on the approval of the study protocol must also be provided in the manuscript.

Magnetic resonance imaging

Experimental design

Design type	Brain MRI for diagnostic purpose
Design specifications	No specific design
Behavioral performance measures	*State number and/or type of variables recorded (e.g. correct button press, response time) and what statistics were used to establish that the subjects were performing the task as expected (e.g. mean, range, and/or standard deviation across subjects).*

Acquisition

Imaging type(s)	structural
Field strength	1.5T
Sequence & imaging parameters	T1 or T2-weighted images
Area of acquisition	whole brain

Diafusion MRI ☐ Used ☒ Not used

Preprocessing

Preprocessing software	No pre-processing used in our study
Normalization	*If data were normalized/standardized, describe the approach(es): specify linear or non-linear and define image types used for transformation OR indicate that data were not normalized and explain rationale for lack of normalization.*
Normalization template	*Describe the template used for normalization/transformation, specifying subject space or group standardized space (e.g. original Talairach, MNI305, ICBM152) OR indicate that the data were not normalized.*
Noise and artifact removal	*Describe your procedure(s) for artifact and structured noise removal, specifying motion parameters, tissue signals and physiological signals (heart rate, respiration).*
Volume censoring	*Define your software and/or method and criteria for volume censoring, and state the extent of such censoring.*

Statistical modeling & inference

Model type and settings	No modeling used in our study
Effect(s) tested	*Define precise effect in terms of the task or stimulus conditions instead of psychological concepts and indicate whether ANOVA or factorial designs were used.*

Specify type of analysis: ☐ Whole brain ☐ ROI-based ☐ Both

Statistic type for inference (See Eklund et al. 2016)	*Specify voxel-wise or cluster-wise and report all relevant parameters for cluster-wise methods.*
Correction	*Describe the type of correction and how it is obtained for multiple comparisons (e.g. FWE, FDR, permutation or Monte Carlo).*

Models & analysis

n/a	Involved in the study
☒	☐ Functional and/or effective connectivity
☒	☐ Graph analysis
☒	☐ Multivariate modeling or predictive analysis
